# Microbe Profile: Vibrio pectenicida: the deadly marine bacteria with strains impacting sea stars and scallops

**DOI:** 10.1099/mic.0.001738

**Published:** 2026-07-15

**Authors:** Paradyse E. Blackwood, Kayla C. King, Amy M. Chan, Alyssa-Lois M. Gehman

**Affiliations:** 1 Department of Zoology, University of British Columbia, Vancouver, BC, Canada; 2 Hakai Institute, Calvert Island, BC, Canada; 3 Department of Microbiology and Immunology, University of British Columbia, Vancouver, Canada; 4 Department of Biology, University of Oxford, Oxford, UK; 5 Department of Earth, Ocean, and Atmospheric Sciences, University of British Columbia, Vancouver, BC, Canada; 6 Institute for the Oceans and Fisheries, University of British Columbia, Vancouver, British Columbia, Canada

**Keywords:** *Vibrio pectenicida*, sea star wasting disease, scallop larvae

## Abstract

*Vibrio pectenicida* is a marine Gram-negative, facultatively anaerobic, rod-shaped bacterium that exists in coastal waters. First described in 1998, these bacteria are associated with deadly disease outbreaks including sea star wasting disease (SSWD) and high mortality in larval scallops in hatcheries. Multiple strain types exist, and *V. pectenicida* A365 is associated with mortality in scallop larvae due to vibrio haemocyte-killer toxin. *V. pectenicida* strain FHCF-3 is associated with SSWD which causes sea stars to ‘melt’ to death. Current research efforts focus on isolating potential new strains to determine if they are causing disease and how this bacterium may influence other marine invertebrates.

## Graphical Abstract

(**a**) *Vibrio pectenicida* is a motile, gram-negative rod-shaped marine bacterium that exists in coastal waters and causes deadly disease outbreaks. First isolated from scallop larvae (type A365), this bacterium causes necrosis and death in these larvae. Recently, *V. pectenicida* FHCF-3 was found to be a causative agent of sea star wasting disease in *Pycnopodia helianthoides* (sunflower sea star). Common disease signs include a deflated body, arm twisting and crossing, body fragmentation, lesions and death. (**b**) A *V. pectenicida* cell adapted from initial discovery by Lambert *et al*., 1998, *International Journal of Systematic Bacteriology* (Lambert *et al*., 1998). Image created in https://www.biorender.com/.

## Taxonomy

Kingdom: bacteria; phylum: *Pseudomonadota*; class: *Gammaproteobacteria*; order: *Vibrionaceae*; family: *Vibrionaceae*; genus: *Vibrio*; species: *pectenicida* (NCBItaxon: 62763 [1]). The species name *pectenicida* reflects its initial isolation and discovery from larval *Pecten maximus* (Great scallop) with the species name meaning scallop-killer [2]. This bacterium species exhibits genetic diversity and has differing strain types (e.g. FHCF-3).

## Properties

*Vibrio pectenicida* is a slow-growing, facultative anaerobic, oxidase-positive, Gram-negative rod-shaped bacterium found throughout coastal waters worldwide [2]. This bacterium is motile due to a single polar flagellum [2]. Pathogenic *V. pectenicida* A365 can produce vibrio haemocyte-killer toxin (VHKT), which destroys bivalve haemocytes, enabling disease development [3]. Bacterial colonies grow well in liquid or solid Marine Broth 2216 and thrive at warmer temperatures (~15–30 °C depending on strain type [2]). Colonies are circular and unpigmented and develop within 24 to 48 h at 20 °C on solid media [2,4]. In liquid culture, *V. pectenicida* FHCF-3 completes all bacterial growth stages in about 5 days (~10–30 °C; pers. obs.).

## Genome

The partial genome/16S rRNA gene of *V. pectenicida* strain A365 (CIP 105190) is 557 bp, and the G+C content is 39–41 mol% [2]. For *V. pectenicida* strain FHCF-3 (JBLZMR000000000), the draft genome is 4,368,354 bp and has 3,903 CDS, 25 rRNA, 92 tRNA and 4 ncRNA genes, with 3 of those sequences having similarity to aerolysin-like toxins that disrupt cellular membranes [5].

## Phylogeny

Phylogenetic analysis revealed that *V. pectenicida* is a distinct member of the *Vibrionaceae* family and the *Vibrio* genus, and this bacterium is in a clade with other *vibrios* such as *Vibrio splendidus*, *Vibrio nereis*, *Vibrio mediterranei* and *Vibrio tapetis*. Specifically, *V. pectenicida* is closely related to *V. tapetis* and *V. splendidus* due to small-subunit rRNA sequences [2,5].

## Key features and discoveries

Marine vibrios, like *V. pectenicida*, are highly pathogenic and can cause various diseases. *V. pectenicida* A365 was originally identified as a strain of interest in French scallop hatcheries (i.e. Argenton, Brittany, France) where it caused 100% mortality in larvae a few days post-exposure [2]. The bacterium produces multiple toxins including VHKT and aerolysin-like toxins that disrupt host tissues and cellular membranes [3,5]. VHKT is a specialized exotoxin that targets and kills marine bivalve immune cells (haemocytes [2,3]). VHKT inhibits metabolic activity and chemiluminescence activity of *P. maximus* haemocytes, provoking rapid cell death [3]. The chemiluminescence assay used tested the production of reactive oxygen intermediates of haemocytes to *V. pectenicida* (i.e. determining if host immune cells mount a defence [3]). Aerolysin-like toxins are pore-forming proteins that act as cytolytic toxins that kill target cells by making holes in their plasma membrane and may contribute to the disease process [5,6]. Sea star wasting disease (SSWD) has caused the mass mortality of 20+ crucial species along the West coast of North America (i.e. Mexico to Alaska), and recently, a novel strain of *V. pectenicida –* FHCF-3 *–* was identified as a causative agent of SSWD in *Pycnopodia helianthoides* [4]. *V. pectenicida* strain 99-46-Y was isolated from a sick large marine clam (geoduck; *Panopea generosa*) in a hatchery in 2000 and causes low levels of mortality in Pacific oyster (*Crassostrea gigas*) larvae [7]. This strain is the reference strain for low virulence *V. pectenicida* [7]. *V. pectenicida* strain 99-46-Y is the closest known relative of *V. pectenicida* FHCF-3 (Average Nucleotide Identity of 98.04%), and both were found in North America [4,7]. Field and lab studies examining SSWD have shown that higher temperatures are a potential trigger for SSWD outbreaks, where areas with warm waters could have increased disease progression, population declines and mortality of sea stars (i.e. *P. helianthoides* and *Pisaster ochraceus* [8,9]). These studies suggest that the temperature interacts with *V. pectenicida*, but future research should be done to directly test how the bacterium reacts to varying temperatures.

## Open questions

We propose a few open questions regarding *V. pectenicida* ecology and evolution.

1. How genetically diverse is this bacterial species? Are these strains/isolates host species specific?
2. Are the two strains A365 and FHCF-3 cross-infective between sea stars and scallops?
3. How is *V. pectenicida* transmitted and how does it infect sea stars?
4. What is the role of environmental factors (e.g. temperature) in shaping the growth and virulence of this bacterium?
5. What is the response of *V. pectenicida* FHCF-3 to environmental conditions (e.g. salinity, temperature, rainfall, etc.)?
6. What is the mechanism of infection of *V. pectenicida* FHCF-3 that causes the disease signs in sea stars (see graphical abstract)?

## Abbreviations

SSWD: sea star wasting disease
VHKT: vibrio haemocyte-killer toxin
